# Annual Direct Medical Costs of Diabetic Foot Disease in Brazil: A Cost of Illness Study

**DOI:** 10.3390/ijerph15010089

**Published:** 2018-01-08

**Authors:** Cristiana M. Toscano, Tatiana H. Sugita, Michelle Q. M. Rosa, Hermelinda C. Pedrosa, Roger dos S. Rosa, Luciana R. Bahia

**Affiliations:** 1 Collective Health Department, Federal University of Goiás, Goiânia, Goiás 74605-050, Brazil; thsugita@gmail.com; 2 Internal Medicine Department, State University of Rio de Janeiro, Rio de Janeiro 20551-030, Brazil; michelleqmrosa@gmail.com (M.Q.M.R.); lucianabahia@gmail.com (L.R.B.); 3 Regional Hospital of Taguatinga, Brasilia 72120-970, Brazil; pedrosa.hc@globo.com; 4 Social Medicine Department, School of Medicine, Federal University of Rio Grande do Sul, Porto Alegre 90035-003, Brazil; roger.srosa@gmail.com

**Keywords:** diabetes mellitus, diabetic foot, cost and cost analysis, health care expenditure, Brazil, foot ulcer, amputation, neuropathy

## Abstract

The aim of this study was to estimate the annual costs for the treatment of diabetic foot disease (DFD) in Brazil. We conducted a cost-of-illness study of DFD in 2014, while considering the Brazilian Public Healthcare System (SUS) perspective. Direct medical costs of outpatient management and inpatient care were considered. For outpatient costs, a panel of experts was convened from which utilization of healthcare services for the management of DFD was obtained. When considering the range of syndromes included in the DFD spectrum, we developed four well-defined hypothetical DFD cases: (1) peripheral neuropathy without ulcer, (2) non-infected foot ulcer, (3) infected foot ulcer, and (4) clinical management of amputated patients. Quantities of each healthcare service was then multiplied by their respective unit costs obtained from national price listings. We then developed a decision analytic tree to estimate nationwide costs of DFD in Brazil, while taking into the account the estimated cost per case and considering epidemiologic parameters obtained from a national survey, secondary data, and the literature. For inpatient care, ICD10 codes related to DFD were identified and costs of hospitalizations due to osteomyelitis, amputations, and other selected DFD related conditions were obtained from a nationwide hospitalization database. Direct medical costs of DFD in Brazil was estimated considering the 2014 purchasing power parity (PPP) (1 Int$ = 1.748 BRL). We estimated that the annual direct medical costs of DFD in 2014 was Int$ 361 million, which denotes 0.31% of public health expenses for this period. Of the total, Int$ 27.7 million (13%) was for inpatient, and Int$ 333.5 million (87%) for outpatient care. Despite using different methodologies to estimate outpatient and inpatient costs related to DFD, this is the first study to assess the overall economic burden of DFD in Brazil, while considering all of its syndromes and both outpatients and inpatients. Although we have various reasons to believe that the hospital costs are underestimated, the estimated DFD burden is significant. As such, public health preventive strategies to reduce DFD related morbidity and mortality and costs are of utmost importance.

## 1. Introduction

Among the various chronic complications that are associated with diabetes mellitus, diabetic foot disease (DFD) is highly frequent, being associated with significant morbidity, mortality, and costs. DFD includes an array of medical conditions, mainly resulting from diabetic peripheral neuropathy and arterial disease, which can lead to foot ulceration. Diabetic foot ulceration may progress with wound infection, osteomyelitis, and, ultimately, amputation. As a result, DFD burden is significant, resulting in major economic consequences for patients, their families, and society.

The global prevalence of DFD varies between 3% in Oceania to 13% in North America, with a global average of 6.4% [1]. It has been estimated in 2015 that each year foot ulcers develop in 9.1 million to 26.1 million people with diabetes worldwide [2]. Furthermore, the incidence of diabetic foot is expected to increase due to the increasing trends in diabetes prevalence and prolonged life expectancy of diabetic patients. In developing countries, 25% of patients with diabetes will develop at least one foot ulcer during their lifetime [3]. More recent evidence suggest that the lifetime incidence of foot ulcers is even higher, between 19% and 34% [4].

Foot ulcers and amputation are more common in low and middle-income countries [5]. However, in such a setting, evidence on the epidemiology of DFD is still lacking. The first comprehensive study of DFD in Brazil, the Brazilian Cooperative Study on Ulcer, Severe Peripheral Neuropathy and Amputation (BRAZUPA), conducted from 2012–2014, evaluated 1055 diabetic individuals in order to gather data on the current situation of foot at risk throughout the nation. A quarter (25.3%) of patients referred previous foot of ulcer, 13.7% amputation (of which 17.3% major amputations), and 5.3% reported history of more than one amputation [6].

A recent review of the economic consequences of diabetic foot lesions indicate that despite the different methods that were used, significant evidence from the literature confirm the substantial economic consequences of diabetic foot lesions [7]. Healthcare costs are five times higher in diabetic individuals with foot ulcers when compared to without foot ulcers [8]. These costs are mainly related to hospitalization, which is more frequent in diabetic individuals with DFD, but also healthcare costs of the clinical management of outpatients with DFD.

Limited evidence is available on the costs of DFD, when considering its whole spectrum and including both hospitalization and outpatient management. Particularly in developing countries, where its burden is higher, such information is important for public health policy makers to advocate for implementation of prevention and treatment recommendations. Healthcare in Brazil is provided by both public and private sectors. Public healthcare services are provided by the National Unified Health System (SUS), which offers free of charge, and universal access to all the population. Healthcare management is decentralized, with municipalities being responsible for managing primary care services [9]. SUS accounts for about 77% of outpatient consultations in the country [10]. Also, a significant proportion of hospitalizations in the country (65.7%) are funded by SUS [11]. The objective of this study was thus to estimate the annual direct medical costs of DFD in the Brazilian Public Health Care System (SUS), when considering both outpatient and inpatient level, in 2014.

## 2. Materials and Methods

### 2.1. Study Design, Site and Population

We conducted a cost-of-illness study, taking a population prevalence-based approach. The analytic horizon was 2014. We took the perspective of the Brazilian Public Health Care System (SUS), and as such, only direct medical costs were considered.

Two methodologies were considered for disease and economic burden estimation, one for outpatients and another for hospitalized patients.

### 2.2. Ethics Approval

The Ethics Committee of Federal University of Goiás in Goiania, Brazil, granted ethical approval for this investigation in October 2014 (# 852 808).

### 2.3. Decision Analytic Model

**Figure 1:**
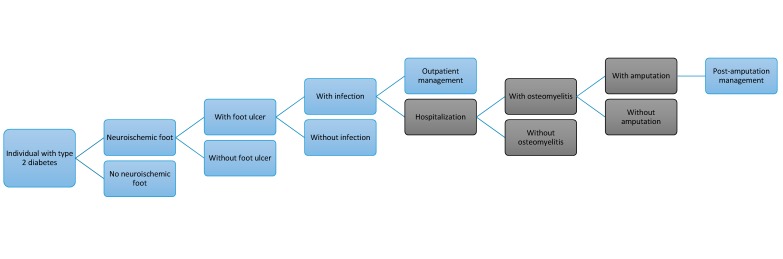
Decision tree diagram for patients with Diabetic Foot Disease and related outcomes. Note: In blue conditions managed in the outpatient setting and in dark grey conditions managed as inpatients.

A decision tree model was constructed to depict the various hypothetical clinical syndromes of DFD and related outcomes, including those managed in the outpatient and inpatient settings (Figure 1). We defined the following possible outcomes while considering the clinical evolution of DFD: neuroischemic foot, active foot ulcer (with/without infection), osteomyelitis with/without amputation, clinical treatment post-amputation.

### 2.4. Model Parameters and Data Sources

Epidemiologic model parameters and sources for base case and sensitivity analysis are listed in Table 1. Population estimates for 2014, considering individuals aged 18 years and older, was obtained from the National Institute of Geography and Statistics [12].

The prevalence of self-reported diabetes was obtained from the 2013 National Health Survey (NHS) [13]. We considered this prevalence for estimating DFD outpatients, while assuming that those who are unaware of DM diagnosis would not refer to primary healthcare center for care.

The original NHS database was analyzed for estimates of prevalence of diabetic individuals with foot ulcer and diabetic individuals with DFD requiring amputation [13]. The estimated prevalence of diabetic individuals with foot ulcer was estimated at 5.27%, and the prevalence of diabetic individuals with DFD requiring amputation was 1.36% when considering a positive response of surveyed individuals to question Q55 “Have you had any of the following diabetes complications?”, in which the respondent specifically answered yes to “foot ulcer” (Q05506), and “amputation of lower extremities” (Q05507), respectively.

We considered the available evidence obtained from national studies in the current literature for values of prevalence of diabetic foot disease among individuals with diabetes (9%) [14], and the proportion of diabetic patients with ulcer with infection (50%) [20].

### 2.5. Outpatient DFD Costs

We used a micro-costing approach based on health resource utilization. A panel of 12 experts in DFD from various reference centers in the country, provided information on healthcare resources utilization for outpatient management of each case. When considering the range of syndromes that were included in the DFD spectrum, costs for the following four well-defined hypothetical DFD cases, occurring in one or two lower extremities, and managed as outpatients were estimated: (1) neuroischemic foot without ulcer, (2) non-infected foot ulcer, (3) infected foot ulcer, and (4) clinical management of amputated patients.

Based on the National Guidelines for DFD management [23,24], we developed a standardized questionnaire in which resource utilization for the following healthcare components were assessed: imaging and laboratory exams; medications; procedures and non-pharmacological therapies (debridement and dressings, in addition to important supplies, such as orthopedic shoes and crutch); and, health professional visits. The questionnaire was piloted and revised based on feedback received from the pilot. Since the centers could have different healthcare access to this specialized treatment, for each healthcare component, a list of treatments included in the guidelines was provided. For the same reason, resources not listed could be added by the expert. In cases where the quantity was not specified, one item per episode was considered. Costs of imaging and laboratory exams; non-pharmacological therapies; and health professional visits were obtained from National Standardized SUS Pricing List [25]. The costs of medications were estimated based on the average price of medication purchased by government [26]. Healthcare resources unit cost were multiplied by quantity, resulting in an estimated average cost for each outpatient hypothetical case.

### 2.6. Hospitalized DFD Patients and Costs

We used a gross-costing methodology based on reimbursement of hospitalized patients. Hospitalizations occurring in SUS are registered into the National Hospitalization Information System (SIH-SUS). For inpatient costs, data of hospitalizations and their costs were collected from SIH-SUS, without personal identification information, which are publicly available online [27].

We considered the main International statistical classification diseases and related health problems, 10th revision (ICD10) codes registered as the cause of hospitalization for analysis [28].

In order to identify the hospitalizations that are related to DFD, we selected medical diagnosis that we considered associated with DFD, categorizing them into two groups: (1) main diagnosis was reported as diabetes (E10, E11, E13, and E14), in which a medical procedure that was related to DFD was performed (i.e., treatment for DFD, surgical stump revision, amputation); and, (2) main diagnosis was reported as any of the following medical conditions related to DFD: neuropathies (G57; 59; 63), ulcers (L97), gangrene (R02), osteomyelitis/osteonecrosis (M86; 87.3; 87.8; 87.9), and amputations (S88; 98) (Table 2).

For diabetes as the main diagnosis in which a medical procedure was performed, we considered all of the patients registered in SIH-SUS. For hospitalizations due to any of the other main diagnosis, assuming that not all of hospitalizations due to this diagnosis are related to DFD, but DM patients with DFD are hospitalized for these conditions more frequently that non-DM individuals, we estimated the etiological fraction of each condition related to DM, using the attributable risk methodology. The risk of presenting a specific medical condition, giving the presence or absence of DM, and the proportion of the population with the disease are combined to calculate the etiological fraction. As such, relative risks estimate for each diagnosis were obtained through systematic literature reviews (Table 2).

We assumed that individuals with diabetes who are unaware of the disease may also be hospitalized due to diabetes or its complications. Brazilian studies with diabetes laboratory confirmation have shown that approximately half of the individuals with diabetes were unaware of the diagnosis [32,33]. As such, for estimation of hospitalized DFD patients, we considered the estimated self-reported diabetes prevalence multiplied by 2, resulting in a national prevalence of diabetes in adults of 12.4%.

All of the hospitalizations due to the above conditions attributable to diabetes were extracted, and the number and costs were estimated. Costs of hospitalizations considered hospitalization charges reported in SIH-SUS, which represent reimbursed values charged for each hospitalization based on Diagnostic Related Groups (DRG), according to standardized national price list [25].

### 2.7. Data Analysis

To estimate the annual national economic burden of outpatient DFD patients, we first estimated the total number of adults with DM in 2014 in Brazil by multiplying the estimated DM prevalence and total Brazilian adult population. We then estimated the number of diabetic outpatients with each of the outcomes being depicted in the decision tree when considering the epidemiologic parameters. The estimated number of patients with each given condition was then multiplied by the estimated average cost per case generating the total economic burden of DFD outpatients in the country.

The burden of hospitalized DFD patients, obtained directly from the SIH-SUS, as previously described, was further incorporated to the overall economic burden of DFD in the country.

Costs were estimated in Brazilian Reais (R$) and then converted to International Dollars (Int$) considering the 2014 purchasing power parity (PPP) (1 Int$ = 1.748 BRL) [34].

### 2.8. Sensitivity Analysis

We performed univariate sensitivity analysis through the variation of the model parameters, as varying estimates are available in the national and international literature. For the following model parameters, we considered lower and higher estimates that were available in the literature as lower and upper bounds for sensitivity analysis: prevalence of neuroischemic foot among DM patients, proportion of DM patients with foot ulcer, proportion of ulcers managed as outpatient vs. inpatient, proportion of foot ulcers progressing to infection, and proportion of patients with neuroischemic foot requiring amputation (Table 1).

## 3. Results

### 3.1. Outpatient Burden and Costs

Assuming that 9.2 million adults have diabetes in Brazil, we estimated that 829,724 (varying from 304,232 to 977,230) of diabetic individuals have neuroischemic foot, of which 43,726 (varying from 3773 to 293,169) present foot ulcers. We estimated that the majority of these patients would be treated as outpatients (*n* = 42,983), and of these, half of them would have an infected ulcer (*n* = 21,492) (Table 3).

We estimated that 11,284 (varying from 3347 to 133,881) individuals are amputated, and thus requiring post-amputation follow up and clinical management.

The estimated average cost of the outpatient management of DFD syndrome was Int$ 343.7 (SD Int$ 104.9) for neuroischemic foot without ulcer, Int$ 408.1 (SD Int$ 287) for non-infected foot ulcer, Int$ 1617 (SD Int$ 1180) for infected foot ulcer, and Int$ 599.8 (SD Int$ 285) for follow-up clinical management of amputated patients.

The resulting total annual direct medical costs of DFD outpatients in the base case was Int$ 335.5 million, varying from Int$ 107.9 million to Int$ 731.6 million in sensitivity analysis. The higher cost share (85%) was for the management of patients with neuroischemic foot without ulcer (Int$ 285.2 million), whereas the costs with infected foot ulcer were estimated at Int$ 24.7 million, non-infected foot ulcer at Int$ 8.7 million, and follow up management of amputated patients at Int$ 6.7 million (Table 4).

### 3.2. Hospitalization Burden and Costs

During 2014, a total of 22,244 patients with diagnosis of diabetes mellitus (ICD10 E10, E11, E13, and E14) in which procedures related to diabetic foot disease were performed were hospitalized. The majority of these hospitalizations were reported as having had diabetic foot treatment (*n* = 12,994), representing 58% of such hospitalizations. As expected, the higher average cost per patient was observed for patients with diabetes in which amputation/disarticulation of lower limb was performed. Total estimated costs for these conditions was Int$ 9.89 million in 2014 (Table 5).

An addition, 28,133 patients were hospitalized due to the other diagnosis of complications related to diabetic foot disease, mainly gangrene in individuals with diabetes (*n* = 15,419). The average hospitalization costs for these combined conditions related to DFD was Int$ 983. Total costs for these conditions was Int$ 17.83 million.

When considering all of the hospitalizations, the total costs of hospitalizations due to DFD in Brazil in 2014 was Int$ 27,721,039.

### 3.3. Overall DFD Costs

The estimated total annual direct medical costs were Int$ 27.7 million for inpatient care and were Int$ 333.5 million for outpatient care, resulting in a total economic burden of Int$ 361 million in 2014.

## 4. Discussion

DFD is responsible for high morbidity and frequent hospital admission in patients with diabetes mellitus, which may result in particularly disabling sequelae, such as lower limb amputation. It is one of the most costly complications of diabetes and can result in an important economic and public health burden, especially in low and middle income countries.

Despite using different methodologies to estimate outpatient and inpatient costs that are related to DFD, this is the first study to assess the overall economic burden of DFD in Brazil, when considering all of its syndromes and both outpatients and inpatients. Although we have various reasons to believe that the hospital costs are underestimated, the estimated DFD burden is significant. We identified significant resource utilization for the management DFD syndromes, particularly related to the large number of individuals on outpatient care. As expected, economic burden was most significantly associated with more severe disease of complex management. In sensitivity analysis, the economic burden of outpatient care varied from Int$ 107.9 million to Int$ 731.6 million, when considering varying parameters from the available literature. The most important parameters driving this variation was the proportion of patients with foot ulcers and requiring amputation. Unfortunately, we were not able to conduct probabilistic sensitivity analysis, as most of the studies that are available did not report uncertainty measures.

Although it is difficult to directly compare our results to other studies in the literature, our data confirms the substantial economic consequences of diabetic foot lesions.

In the United States, a total of $176 billion is spent annually on direct costs for diabetes care, with as much as one third of this expenditure being related to lower-extremity conditions [35].

Our results are in line with those reported in a recent study conducted in Peru, also a Latin American developing country. With an estimated population of individuals with diabetes of 942,000, 10% of the estimated diabetic population in Brazil, estimated direct medical costs for the management of diabetic foot were $70 million in 2012 [36]. A more recent study in Trinidad and Tobago in the Caribbean estimated the costs of economic burden of diabetic foot infection at $85 million, or 0.4% of their GDP [37].

We estimated DFD outpatient costs using similar methodology, as used by Cavanagh et al. (2012) when assessing treatment costs in five different countries (Chile, China, India, Tanzania, and the United States), for two hypothetical foot ulcers at the extreme ends of the complexity spectrum [38]. As also reported by Cavanagh, our results also demonstrated that treatment costs were higher in more severe DFD syndromes.

Our estimated direct costs for outpatient management of infected ulcers (Int$ 1617) is significantly lower than the reported in a review of health-economic consequences of diabetic foot lesions (~$17,500 in 1998) [7]. Likewise, our reported costs for lower-extremity amputations (Int$ 1000) was significantly lower than the $30,000 reported by Tennvall et al. [7]. However, of worth noting is the fact that the review included only studies conducted in developed high-income countries.

In 2014, the Brazilian GDP was Int$ 3.307 trillion [39], of which 8.3% (Int$ 274.48 billion) was spent in health [40]. Of these, public health care expenditures represent 42.5% (Int$ 116.73 billion) [41]. Our study estimated that the annual direct medical costs of DFD in 2014 was Int$ 361 million, which denotes 0.31% of public health expenses for this period. When looking at SUS hospitalization expenditures only, the federal government spent Int$ 7.414 billion in 2014 [42]. In our study, hospitalizations for DFD accounted for 0.37% of this amount.

Overall, recent studies on the economic burden of DFD in Brazil mainly focused on hospitalizations of patients with DFD [43,44,45,46]. All except one [44] report average hospitalization costs for specific conditions related to DFD, i.e., amputation. Using, as we did, SUS reimbursement gross costing methodology, Milman et al. (2001) [47] evaluated 23 patients in a single hospital, of which 65% evolved to amputation, reporting an average costs of R$ 1004 (Int$ 1369). Also, using similar methodology, Oliveira et al. (2014) [46] reported an average cost of R$2866 (Int$ 1838), for 40 patients with DFD hospitalized in the SUS in 2012 with very high variations of costs among patients. Although this is almost twice as our average estimated hospitalization costs (Int$ 983), the small sample size and restricted in one single hospital in the country limit the external validity of these findings. Finally, Haddad et al. [45] also evaluated 21 diabetic patients that were hospitalized for amputation in a single hospital in 2006, reporting an average cost of R$ 4621 per patient (Int$ 4208), or almost four times higher our average costs. In all of these studies, the limited external validity may suggest that those estimates, although higher, are not representative of the totality of DFD hospitalizations occurring in the SUS nationwide.

Comparisons of results from various health-economic studies are challenged by methodological variations, including study design, perspective and time frame; data source; patient population; costing methods; types of costs considered; types of foot lesions considered; and, finally, health care system structure, settings, treatment practices, and reimbursement systems that are in place. Furthermore, the external validity of costing studies is limited. Finally, some studies lack information about the year of costing, exchange rate, and type of costs included [7].

We believe our hospitalization costs are underestimated for several reasons. First, we considered hospital charges, which is known to be significantly lower than costs as measured by micro-costing methods [7]. While international studies report that hospital costs may be only 70–80% of charges billed [7], it has been estimated that the actual cost of the hospitalization in Brazil is, on average, seven times higher than the amount reimbursed by SUS [48]. To this account, a recent study compared hospitalization costs of DFD hospitalizations within SUS in Brazil, reporting that hospitalization costs estimated by micro-costing were as much as 10 times higher than reimbursement costs [48]. Second, coding for DFD related conditions is not straightforward, and thus we may have missed hospitalizations due to DFD, but with codes for hospitalization diagnosis different that the ones we considered as related to DFD.

Despite the wide variation in selected model parameters based on the available evidence, we selected for the case base values that were either from the national literature or from nation surveys or secondary data. We were fortunate to have estimates from a recent National Health Survey for both proportion of DM patients with foot ulcers and amputation. When we contrast the estimated number of patients requiring outpatient follow-up after amputation (11,284) based on modeling considering this parameter with the total number of individuals hospitalized for amputation as observed in the SIH-SUS (11,500), it is reassuring to see very similar figures, despite being estimated by different methods.

Nonetheless, other model parameters as prevalence of neuroischemic foot and proportion of infected ulcers were obtained from small regional studies, and may not express national prevalence.

When considering all of the above, it is not surprising that when comparing our results to the only modelling study conducted in Brazil in which economic burden of DFD hospitalizations only are estimated [44], our estimated hospitalization cost (Int$ 27.7million) is almost 10 times lower that that reported by Rezende et al. (2010) (USD 264 million). If one assumes that our hospitalization costs might be underestimated by a factor of 10, as some evidence suggests, the reported figures would be equivalent.

Finally, it is worthwhile mentioning that as we considered the perspective of the Brazilian Public Health Care System, our burden estimates include only direct medical costs. Non-medical costs, productivity loss, costs with orthoses and prostheses, and home care and social service costs for patients who have undergone lower-extremity amputation, were not included in this study. As such, the overall economic burden of DFD is even more significant in Brazil.

Comprehensive diabetic foot care, based on prevention, education and a multi-disciplinary team approach, may reduce foot complications and amputations by up to 85% [49]. Recent initiates in Brazil have contributed to raise awareness towards DFD in the country, such as the “Save the Diabetic Foot Project” in the early 90’s [50]. Nonetheless, the organization of services for the prevention and care of DFD is still sub-optimal. This is the first nationwide study to estimate the economic burden of DFD when considering its full spectrum and including both outpatients and inpatients. These results are of use for raising awareness and promoting evidence based decision making on the implementation of public health policies that are aimed at diabetic foot care.

## 5. Conclusions

Little evidence is available on the economic burden of DFD in Brazil. We identified significant resource utilization for the outpatient management DFD syndromes, particularly related to healthcare personnel. Costs were higher for more severe disease of complex management. DFD imposes a significant health and economic burden to the Brazilian Healthcare System, emphasizing the need for health policies that are targeting its improved prevention and care.

## Figures and Tables

**Table 1:** Epidemiology model parameters—Base case and sensitivity analysis.

Model Parameters	Base Case		Sensitivity Analysis			
	Value	Reference	Lower	Reference	Upper	Reference
Total adult population, 2014	148,696,000	[12]				
Prevalence of self-reported diabetes (%)	6.2	[13]				
Prevalence of neuroischemic foot among DM ^#^ patients (%)	9	[14]	3.3	[15]	10.6	[14]
Proportion of DM ^#^ patients with ulcers (%)	5.27	[13]	1.24	[15]	30	[16]
Ulcers managed as outpatients * (%)	98.31	[17]	65.2	[18]	99.7	[19]
Non-infected foot ulcer (%)	50	[20]	88	[21]	44.5	[22]]
Infected foot ulcer (%)	50	[20]	12	[21]	55.5	[22]
Ulcers managed as inpatients	1.69	[17]	34.8	[18]	0.3	[19]
Amputation (%)	1.36	[13]	1.1	[15]	13.7	[6]

^#^ DM = diabetes mellitus; * Estimated from the frequency of hospitalized treated infected ulcers.

**Table 2:** Causes of hospitalization and procedures related to diabetic foot disease, as coded by ICD-10 codes, relative risk and source.

Diabetes Mellitus *			
E10	Insulin-dependent diabetes mellitus		
E11	Non-insulin-dependent diabetes mellitus		
E13	Other specified diabetes mellitus		
E14	Unspecified diabetes mellitus		
**Complications Related to Diabetic Foot Disease**		Relative Risk	Ref.
G57	Mononeuropathies of lower limb	1.97	[29]
G59	Mononeuropathy in diseases classified elsewhere	1.97	[29]
G63	Polyneuropathy in diseases classified elsewhere	1.97	[29]
L97	Non-pressure chronic ulcer of lower limb, not elsewhere classified	1.97	[29]
M86	Osteomyelitis	5.8	[30]
M87.3	Other secondary osteonecrosis	5.8	[30]
M87.8	Other osteonecrosis	5.8	[30]
M87.9	Unspecified osteonecrosis	5.8	[30]
R02	Gangrene, not elsewhere classified	10.9	[31]
S88	Traumatic amputation of lower leg	6.4	[31]
S98	Traumatic amputation of ankle and foot	19.4	[31]

* When the following procedures were conducted during hospitalization: Wound healing with or without debridement, Ulcer debridement, Diabetic foot treatment, Treatment of other vasculopathies, Treatment of long-term care patient due to osteomuscular disease, Treatment of polyneuropathies, Intraluminal angioplasty of peripheral vessels, Revascularization of femoral-popliteal or other distal arteries, Amputation/disarticulation of lower limbs/foot/tarsus/tallus, Surgical revision of amputation stump, Femoral/Hip disarticulation.

**Table 3:** Estimated number of diabetics and outpatients with diabetic foot diseases. Base-case and sensitivity analysis. Brazil, 2014.

DFD Condition	Number of Individuals		
	Base Case	Sensitivity Analysis	
Individuals with diabetes mellitus	9,219,152	Lower	Upper
DM patients with neuroischemic foot	829,724	304,232	977,230
DM patients with ulcers	43,727	3773	293,169
Patients with ulcers managed as outpatients	42,984	2460	292,290
Non-infected foot ulcer	21,492	2165	130,069
Infected foot ulcer	21,492	295	162,221
Patients amputated requiring follow up	11,284	3347	133,880

**Table 4:** Estimated annual direct medical costs of diabetic foot disease (DFD) outpatients. Base-case and sensitivity analysis. Brazil, 2014.

DFD Condition	Value in Int$		
	Base Case	Sensitivity Analysis	
		Lower	Upper
Neuroischemic foot without ulcer	285,197,635	104,572,466	335,899,436
Non-infected foot ulcer	8,771,482	833,410	53,085,829
Infected foot ulcer	34,752,923	477,287	262,319,121
Patients amputated requiring follow up	6,767,704	2,007,089	80,294,609
Total outpatient DFD costs	335,489,743	107,940,251	731,598,996

**Table 5:** Number, average cost and total costs of hospitalizations due to Diabetic Foot Disease. Brazil, 2014.

Diabetes Mellitus (E10, E11, E13, E14) *		**Number (*n*)**	**Average Hospitalization Cost (Int$)**	**Total Hospitalization Cost (Int$)**
	Diabetic foot treatment	12,994	306.1	3,976,997
	Amputation/disarticulation of lower limbs	3318	1097.5	3,641,671
	Amputation/disarticulation of foot/tarsus	1820	354.3	644,869
	Surgical revision of lower limb amputation stump	209	388.8	81,266
	Amputation/disarticulation of toe	3817	400.3	141,688,219
	Surgical revision of toe amputation stump	86	224.5	19,310
Sub-Total		22,244	444.7	9,892,152
**Complications related to Diabetic Foot Disease**				
Ulcer	L97—Non-pressure chronic ulcer of lower limb, not elsewhere classified	3545	527.5	1,870,290
	R02—Gangrene, not elsewhere classified	15,419	668.9	10,313,991
Neuropathy	G57—Mononeuropathies of lower limb	29	498.7	14,404
	G59.0—Mononeuropathy in diseases classified elsewhere	5	163.6	817
	G63—Polyneuropathy in diseases classified elsewhere	276	382.3	105,460
Osteomyelitis	M86—Osteomyelitis	5849	554.1	3,240,765
	M87.3—Other secondary osteonecrosis	177	2710.2	480,326
	M87.8—Other osteonecrosis	125	2530.9	315,405
	M87.9—Unspecified osteonecrosis	168	1346.5	226,575
Amputation	S88—Traumatic amputation of lower leg	439	1055.6	463,567
	S98—Traumatic amputation of ankle and foot	2100	378.3	794,283
Sub-Total		28,133	983.3	17,825,887
